# GC/MS Profiling, Antibacterial, Anti-Quorum Sensing, and Antibiofilm Properties of *Anethum graveolens* L. Essential Oil: Molecular Docking Study and In-Silico ADME Profiling

**DOI:** 10.3390/plants12101997

**Published:** 2023-05-16

**Authors:** Emira Noumi, Iqrar Ahmad, Mohd Adnan, Abderrahmen Merghni, Harun Patel, Najla Haddaji, Nouha Bouali, Khulood Fahad Alabbosh, Siwar Ghannay, Kaïss Aouadi, Adel Kadri, Flavio Polito, Mejdi Snoussi, Vincenzo De Feo

**Affiliations:** 1 Department of Biology, College of Science, University of Hail, P.O. Box 2440, Hail 81451, Saudi Arabia; mo.adnan@uoh.edu.sa (M.A.); najla_haddaji@yahoo.fr (N.H.); nouhabouali82@gmail.com (N.B.); k.alabosh@uoh.edu.sa (K.F.A.);; 2 Laboratory of Genetics, Biodiversity and Valorization of Bio-Resources (LR11ES41), Higher Institute of Biotechnology of Monastir, University of Monastir, Avenue Tahar Haddad, Monastir 5000, Tunisia; 3 Department of Pharmaceutical Chemistry, Prof. Ravindra Nikam College of Pharmacy, Gondur, Dhule 424002, Maharashtra, India; ansariiqrar50@gmail.com; 4 Laboratory of Antimicrobial Resistance LR99ES09, Faculty of Medicine of Tunis, University of Tunis El Manar, Tunis 1068, Tunisia; abderrahmen_merghni@yahoo.fr; 5 Division of Computer Aided Drug Design, Department of Pharmaceutical Chemistry, R. C. Patel Institute of Pharmaceutical Education and Research, Shirpur 425405, Maharashtra, India; hpatel_38@yahoo.com; 6 Department of Chemistry, College of Science, Qassim University, P.O. Box 6688, Buraidah 51452, Saudi Arabia; s.ghannay@qu.edu.sa (S.G.);; 7 College of Science and Arts in Baljurashi, Al Baha University, P.O. Box 1988, Albaha 65527, Saudi Arabia; 8 Laboratory of Plant Biotechnology Applied to Crop Improvement, Faculty of Sciences of Sfax, University of Sfax, Sfax 3000, Tunisia; 9 Department of Pharmacy, University of Salerno, Via Giovanni Paolo II, 132, 84084 Salerno, Italy

**Keywords:** *Anethum graveolens* L., essential oil, chemical composition, pathogenic bacteria, antibiofilm, pharmacokinetics, molecular docking

## Abstract

*Anethum graveolens* L. has been known as an aromatic, medicinal, and culinary herb since ancient times. The main purpose of this study was to determine the chemical composition, antibacterial, antibiofilm, and anti-quorum sensing activities of the essential oil (EO) obtained by hydro-distillation of the aerial parts. Twelve components were identified, representing 92.55% of the analyzed essential oil. Limonene (48.05%), carvone (37.94%), cis-dihydrocarvone (3.5%), and trans-carvone (1.07%) were the main identified constituents. Results showed that the obtained EO was effective against eight bacterial strains at different degrees. Concerning the antibiofilm activity, limonene was more effective against biofilm formation than the essential oil when tested using sub-inhibitory concentrations. The results of anti-swarming activity tested against *P*. *aeruginosa* PAO1 revealed that *A. graveolens* induced more potent inhibitory effects in the swarming behavior of the PAO1 strain when compared to limonene, with a percentage reaching 33.33% at a concentration of 100 µg/mL. The ADME profiling of the identified phytocompounds confirms their important pharmacokinetic and drug-like properties. The in-silico study using molecular docking approaches reveals a high binding score between the identified compounds and known target enzymes involved in antibacterial and anti-quorum sensing (QS) activities. Overall, the obtained results highlight the possible use of *A. graveolens* EO to prevent food contamination with foodborne pathogenic bacteria.

## 1. Introduction

The use of plants in alternative medicine has increased during the last 25 years [1]. Medicinal and aromatic plants (MAPs) are a rich reservoir of bioactive molecules, able to promote health and be used as drugs [2,3,4,5]. The overuse of antibiotics to treat infectious diseases contributed to the emergence of multidrug-resistant bacterial strains. This fact generated a renewed interest in plant therapy medicine, which has become interesting in recent decades [6]. The beneficial effects of many plants, essentially their antimicrobial potential, are largely studied around the world [7]. Essential oils and plant extracts are used to replace synthetic antioxidants and antimicrobial agents in the food and pharmaceutical industries and in phytotherapy. In fact, Shan et al. [8] reported that 46 extracts from medicinal plants and spices possessed antibacterial activity against bacteria isolated from contaminated food preparations.

Essential oils contain highly active antimicrobial molecules such as thymol, carvacrol, terpenoids, and eugenol. Furthermore, many researchers have investigated the mode of action of essential oils [9].

*Anethum graveolens* L., commonly known as dill, is a medicinal plant from the family Apiaceae, native to the Mediterranean region, southeastern Europe, and central and southern Asia. Currently, it is cultivated widely throughout the world [10,11,12,13]. Dill is largely used in the food industry for sauces, salads, and seafood. It has been reported that *Anethum graveolens* has antimicrobial, anti-inflammatory, analgesic, diuretic, hypotensive, antispasmodic, smooth muscle relaxant, antiemetic, and laxative effects. Moreover, it is used as an anti-convulsant and anti-emetic [14,15,16]. It was used as a remedy for gastrointestinal disorders [16]. Previous studies showed that *A. graveolens* contains essential oils [17], moisture (8.39%), proteins (15.68%), carbohydrates (36%), fiber (14.80%), ash (9.8%), furanocoumarin, polyphenols, and mineral elements (potassium, calcium, magnesium, phosphorous, and sodium) [18]. Dill is also rich in vitamin A and niacin [19].

Moreover, dill seed essential oil is rich in carvone (20–60%) [20], limonene, α-phellandrene, α-pinene, α-terpinene, apiole, dill apiole, 1,8-cineole, dihydrocarvone, and *p*-cymene [17]. *A. graveolens* seeds are commonly used in Serbian food preparations [21]. The antimicrobial potential of *A. graveolens* EO has been largely studied [22]. It was demonstrated that the flavonoids and the terpenoids exhibit effectiveness against the pathogens [23]. Researchers are interested in the isolation and characterization of chemical constituents to study their biological activities. Nowadays, molecular docking approaches have become an important tool for drug discovery [24]. The phytochemical screening of plant compounds can inform us about the biological effects of plant extracts and essential oils (EO); however, the phytoconstituent responsible for this action is still unknown [25]. Thus, in silico docking studies are essential to understanding the affinity and interaction between the identified compounds and target proteins [26,27,28,29]. 

The purpose of this work was to determine the active compounds in *A. graveolens* EO by using the GC-MS technique to further study its antibacterial and anti-biofilm activities against several Gram positive and Gram negative foodborne pathogenic bacteria. The ability of dill EO and its main compound to attenuate the quorum sensing system was also tested using *Pseudomonas aeruginosa* and *Chromobacterium violaceum* strains. Moreover, the draggability and pharmacokinetic properties of *A. graveolens* have been evaluated using ADME profiles and molecular docking approaches.

## 2. Results

### 2.1. Chemical Composition of A. graveolens EO

The chemical composition of *A. graveolens* EO identified by using the GC/MS technique is listed in Table 1. Twelve components were identified, representing 92.55% of the analyzed essential oil. In fact, the results obtained highlighted that *A. graveolens* EO was rich in limonene (48.05%), carvone (37.94%), dihydro carvone cis (3.5%), and trans-carvone (1.07%).

**Figure 1:**
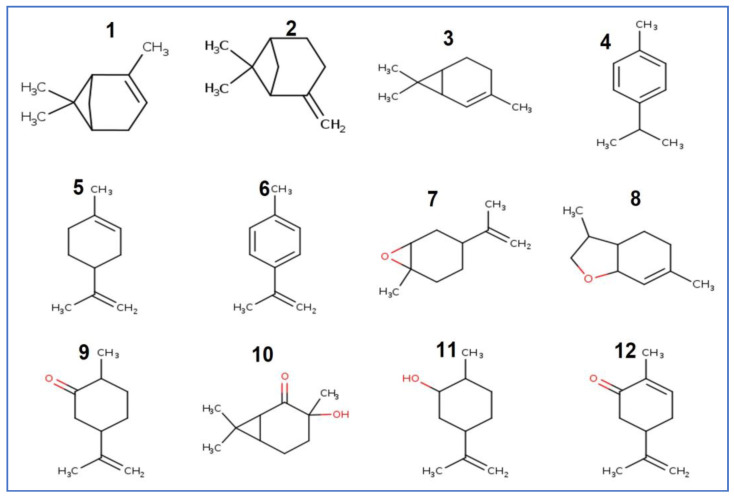
Chemical structure of the *A. graveolens* EO identified compounds. All compound names are listed in Table 1.

The total number of identified compounds in the EO of *A. graveolens* is divided into several chemical groups that are essentially cyclic monoterpenes, such as limonene, pinene, and cymene. The terpenoids group is represented by carvone. The chemical structures of all identified compounds are represented in Figure 1 below.

### 2.2. Antibacterial Potential of A. graveolens EO and Limonene

The antibacterial effect of *A. graveolens* EO and its main compound, limonene, was first assessed by the determination of the growth inhibition zone (GIZ) on Mueller Hinton (MH) agar plates. The obtained results are summarized in Table 2. In fact, *A. graveolens* EO showed a potent antibacterial effect against all tested microorganisms (except for *P. aeruginosa* PAO1) as compared to limonene, with GIZ ranging from 10 ± 0.01 to 28.5 ± 0.71 mm. The main compound was found to be active only against three pathogenic strains (*L. monocytogenes*, *S. aureus,* and *S. enterica*) with ZI > 10 mm. The results of minimal inhibitory concentrations (MICs) and minimal bactericidal concentrations (MBCs) showed that both tested agents exerted a bacteriostatic effect (MBC/MIC > 4) against all tested strains.

Using the microdilution technique (Table 2), MIC values of *A. graveolens* EO ranged from 0.048 to 0.097 mg/mL for bacterial strains, and MBC values ranged from 12.5 to >50 mg/mL. For limonene, the main component identified in *A. graveolens* EO, all MIC values were about 0.048 mg/mL, and the MBCs were about 50 mg/mL. Interestingly, a weak concentration of *A. graveolens* EO and limonene (0.048 mg/mL) exhibited an inhibitory effect against Gram-positive and Gram-negative pathogenic bacteria used in this study. Moreover, the tested essential oil showed bacteriostatic activity against almost all tested foodborne pathogenic bacteria, as the calculated values of MBC/MIC ratios were higher than 4 [30,31]. 

### 2.3. Adhesive Properties of Bacterial Strains

**Figure 2:**
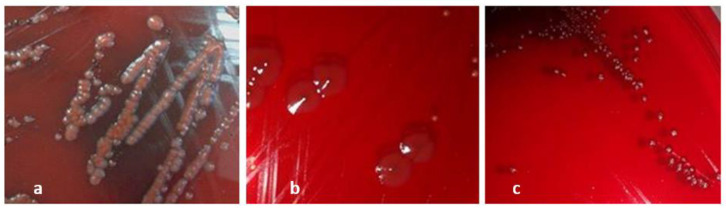
Different morphotypes obtained on CRA plates based on the color of the colonies. (**a**): negative slime producer, (**b**,**c**): positive slime producer.

The results of slime production on Congo red agar (CRA) plates revealed that five (62.5%) strains were able to produce exopolysaccharides, displaying black or red colonies with black center colonies (Figure 2, Table 3). 

Additionally, among tested strains, four out of eight bacteria were moderately adherent to glass tubes (noted 2+), and two strains were highly adherent (noted 3+; Table 3). Regarding the bacterial adhesiveness on polystyrene surfaces, evaluated with the CV staining assay, our results showed that all strains are moderate biofilm producers (0.1 ≤ OD_570_ < 1), except for *S. aureus* ATCC 6538, which was found to be a highly biofilm producer (OD_570_ ≥ 1) (Table 3).

**Figure 3:**
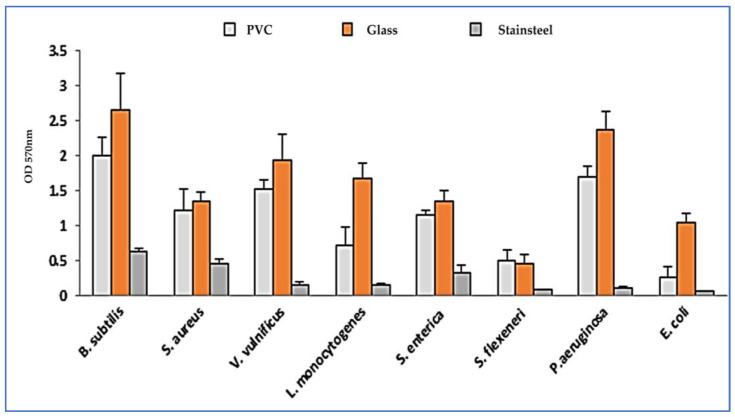
Biofilm formation ability of pathogenic bacteria on various materials. Error bars indicate SD (± standard deviation) of three independent experiments.

The biofilm formation on various abiotic surfaces revealed that most tested bacteria are able to produce biofilm structures on glass and polyvinyl chloride (PVC) materials. However, they showed low-grade biofilm formation on the stain-steel surface (OD_570_ < 1). In addition, the tested Gram-positive bacteria showed more potent biofilm formation abilities as compared to the Gram-negative ones (Figure 3). 

### 2.4. Anti-Adhesion and Anti-Biofilm Formation Activities

**Figure 4:**
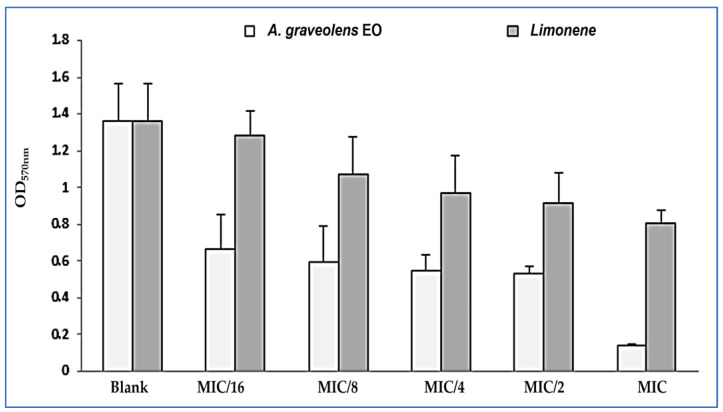
Anti-adhesion activities of *A. graveolens* EO and limonene. Error bars indicate SD (± standard deviation) of three independent experiments.

The anti-adhesion effect of *A. graveolens* EO and its main compound (limonene) was also tested. After treatment with different sub-inhibitory concentrations (1/16 × MIC to 1 × MIC) bacterial adhesion was more affected by EO than limonene, since at a concentration of MIC/16 (0.003 mg/mL), *A. graveolens* EO exhibited an anti-attachment effect in comparison with the untreated bacteria (control). While MIC/2 of limonene has anti-adhesion activity against the tested strains (Figure 4).

**Figure 5:**
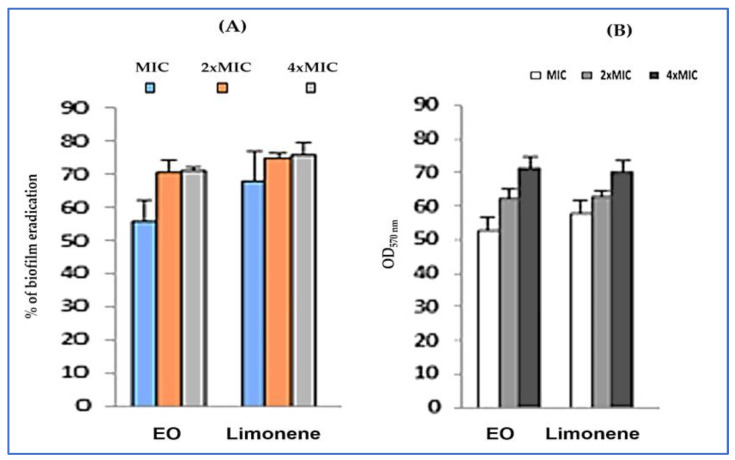
Anti-biofilm activities of *A. graveolens* EO and limonene on polystyrene (**A**) and glass (**B**) surfaces. Error bars indicate SD (± standard deviation) of three independent experiments.

The anti-biofilm effect of *A. graveolens* EO and limonene on mature biofilm (48 h) subjected to different concentrations (ranging from MIC to 4 × MIC) revealed that both tested agents showed high biofilm eradication on polystyrene and glass surfaces, with percentage reduction exceeding 50% at low concentrations (1 × MIC) (Figure 5). Additionally, limonene was more effective against biofilm formation than the essential oil, showing the highest percentage of biofilm eradication.

### 2.5. Anti-Quorum Sensing Activities of the Tested Agents

#### 2.5.1. Anti-Swarming Activity

The results of anti-swarming activity tested against *P*. *aeruginosa* PAO1 revealed that *A. graveolens* induced a more potent inhibitory effect in the swarming behavior of PAO1 strains when compared to its mean compound limonene, with a percentage reaching 33.33% at a concentration of 100 µg/mL (Table 4).

#### 2.5.2. Violacein Inhibition Assay

In qualitative analysis, MIC values of *A. graveolens* EO and limonene showed an inhibition of 67.52 % in violacein production. This inhibition was about 57.91% at MIC/2 of *A. graveolens* EO and limonene. A concentration of 0.3125 mg/mL (MIC/32) of the EO inhibited 23.66% of the bacteria’s growth (Table 5).

**Figure 6:**
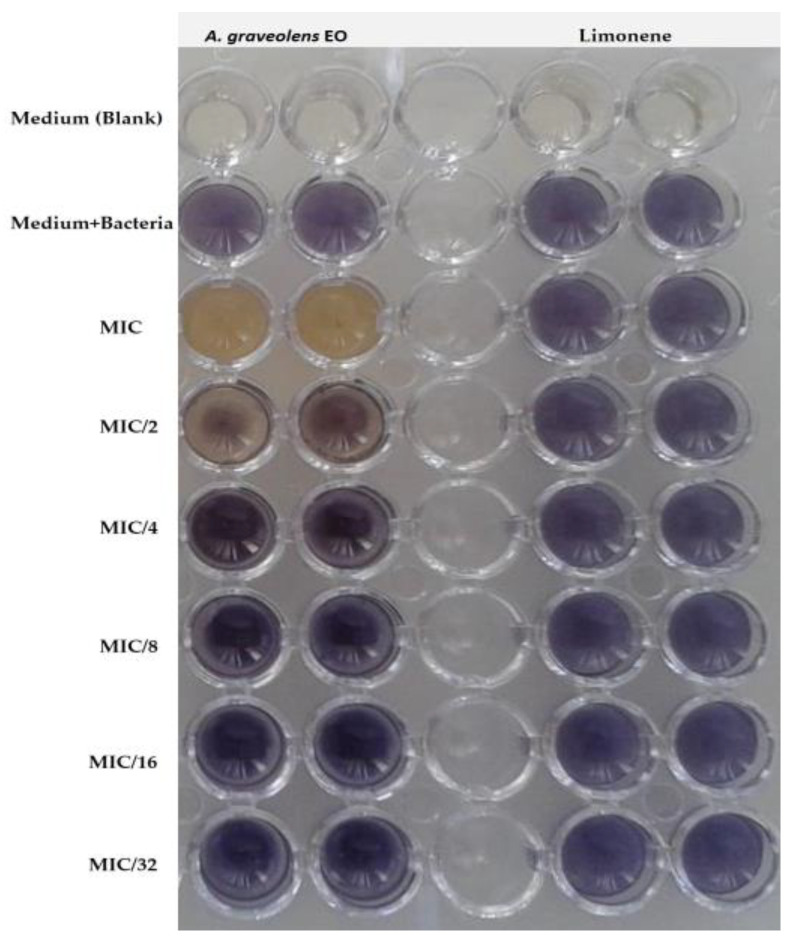
Effect of different MIC values of *A. graveolens* EO and limonene on violacein inhibition in *C. violaceum* ATCC 12472.

Limonene was able to inhibit violacein production by 6.33% until the concentration of 0.156 mg/mL (MIC/8) (Table 5, Figure 6).

### 2.6. Draggability and Pharmacokinetic Properties of A. graveolens Main Compounds 

**Figure 7:**
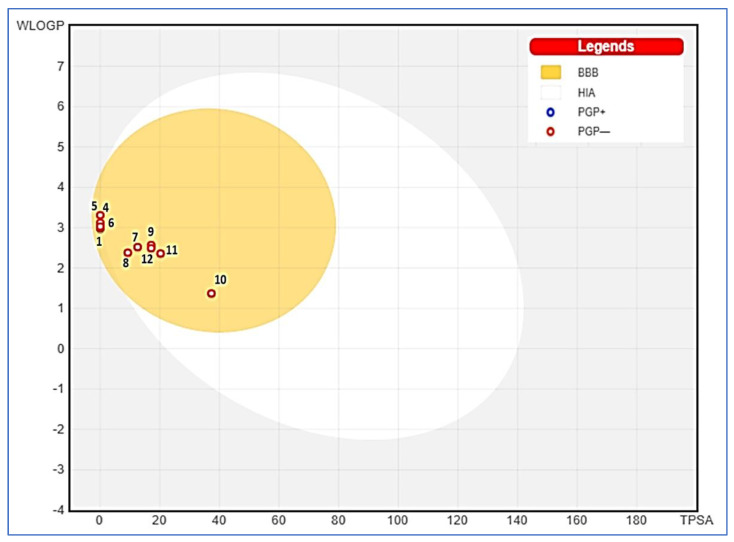
Boiled-egg model of *A. graveolens* studied compounds. The names of the compounds are listed in Table 1.

The ADME properties of the twelve identified compounds were studied (Table 6). Interestingly, the studied compounds displayed a nil alert. Using the results from the boiled egg, β-pinene and δ-2-carene were out of the obtained model (Figure 7). Moreover, all compounds had acceptable consensus Log Po/w ranging from 1.56 to 3.5. In addition, Lipinski’s rule was confirmed, and good gastro-intestinal absorption, lipophilicity, and bioavailability scores (0.55–0.58) were reported. In addition, all selected compounds exhibited good topological polar surface area values (TPSAs) lower than 125 Å^2^, suggesting that they are expected to be orally absorbed. 

**Figure 8:**
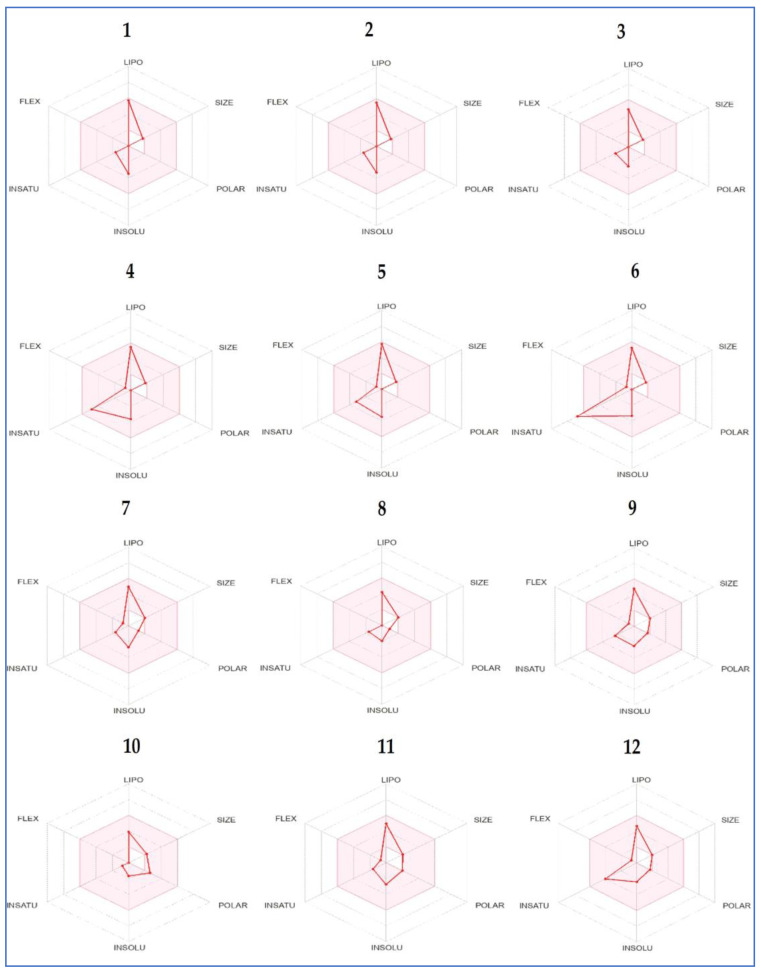
Bioavailability polygons of *A. graveolens* identified compounds based on their physicochemical parameters using ADMET properties. Names of the compounds are same as in Table 1.

The drug-like behavior of all identified compounds represented by the bioavailability radar showed that they fit within the pink area of the polygon (Figure 8).

Similarly, all compounds were blood–brain barrier (BBB) permeable. Interestingly, compounds 1 (α-pinene), 2 (β-pinene), and 5 (limonene) inhibited four cytochrome P450 isoenzymes (CYP2C9). The compounds 4 and 6 (ρ-cymene and meta-cymene) were able to inhibit four cytochrome P450 isoenzymes (CYP2D6). All selected compounds exhibited negative Log Kp values (skin permeability) ranging from −3.89 to −6.41, highlighting their suitability as good compounds to be delivered transdermally. All these results are summarized in Table 6.

### 2.7. Molecular Docking Study

The study of molecular docking delivers an understanding of molecular binding affinities and the binding approach of the phyto-constituents within the targeted protein. The molecular docking study was carried out against five different receptors, namely the human peroxiredoxin 5 receptor (PDB: 1HD2), TyrRS from *S. aureus* (PDB: 1JIJ), type IIA topoisomerase from *S. aureus* (PDB: 2XCT), and the LasR protein receptor of *P. aeruginosa* (PDB ID: 2UV0 and 3IX3), to determine the binding affinities. The outcomes of the docking studies are mentioned concisely in Table 7. As shown in Table 7, all compounds had negative binding energies (ranging from −3.52 to −7.07 kcal/mol) with the different target receptors, with dihydrocarvone cis showing the most promising docking score of all the receptors investigated. On the other hand, caranone trans (−5.622 kcal/mol) and meta-cymene (−6.875 kcal/mol) have promising docking scores on the 1JIJ and 2UV0 receptors, respectively. The binding affinity of 1HD2 protein, which ranges from −3.03 to −5.105 kcal/mol, is less than that of the target co-crystallized ligand, benzoic acid, which has the highest binding affinity (−7.245 kcal/mol). In the 3IX3 target, α-pinene, β-pinene, δ-2-carene, ρ-cymene, meta-cymene, dill ether, trans-caranone, and dihydro carveol (neoiso) have the highest docking score, which is better than the co-crystalized inhibitor (−6.05 kcal/mol).

## 3. Discussion

In this work, we used the GC/MS technique to identify the active compounds in *A. graveolens* EO. The main identified phytoconstituents were limonene (48.05%), carvone (37.94%), cis-dihydro carvone (3.5%), and trans-carvone (1.07%). In fact, it was previously demonstrated that the chemical composition of *A. graveolens* EO varies according to the plant organ, the time of the collection of the organ plant, the geographic origin, and seasonal and climatic factors [32,33]. Previous studies have demonstrated that the fruits of *A. graveolens* are rich in carvone (30–60%), limonene (33%), and α-phellandrene (20.61%) [34,35]. Al-Ma’adhedi et al. [32] and Singh et al. [33] demonstrated that the major compounds of *A. graveolens* oil are d-limonene (45%) and D-carvone (23.1%). Dill essential oil is also known to contain eugenol, anethole, flavonoids, coumarins, triterpenes, and phenolic acids. Another study confirmed that *A. graveolens* EO extract from seeds is rich in limonene and carvone [36]. Apart from carvone, limonene (5.1%), cis-dihydrocarvone (3.0%), trans-dihydrocarvone (2.7%), cis-carveol (1.8%), and trans-carveol (1.4%), all other components of oil were identified in much lower concentrations [21]. 

Dill was used in food and drugs. Several studies focused on the biological activities of compounds present in EO or/and extracts. It has been demonstrated that A. graveolens has many medicinal uses: antibacterial, antispasmodic, antitumor, digestive, carminative, and could be used as a cardioprotective agent [37]. The most important activity studied was the effect on pathogenic microorganisms due to their resistance to commercialized antibiotics [38]. Essential oils and plant extracts can be used as a solution for the treatment of many infectious diseases [39]. Dill is a rich plant in chemical constituents with several biological effects, especially against multidrug-resistant microorganisms [37]. A relationship between the chemical composition of dill EO and its antimicrobial properties has been proven, and it can be used in the pharmaceutical and food industries as a natural additive [40]. Our results showed that *A. graveolens* EO showed an important antibacterial effect against foodborne pathogenic bacteria compared to limonene using both solid and liquid methods. The MIC values of *A. graveolens* EO ranged from 0.048 to 0.097 mg/mL for bacterial strains, and the MBCs ranged from 12.5 to >50 mg/mL. The results of MICs and MBCs showed that both tested agents (EO and limonene) exerted a bacteriostatic effect (MBC/MIC > 4) against all tested strains [30,31]. 

In our study, commercial dill seed essential oil, rich in limonene (48.05%) and carvone (37.94%), showed antimicrobial activity against almost all tested Gram positive and Gram-negative bacteria. The essential oil of *A. graveolens* seeds exerted antimicrobial activity against *Staphylococcus aureus*, *Bacillus cereus*, *Enterococcus faecalis*, *Listeria monocytogenes*, *Escherichia coli*, *Yersinia enterocolitica*, *Salmonella typhimurium*, *Shigella flexneri*, *Pseudomonas aeruginosa*, and *Mycobacterium* [37,39]. It was demonstrated that D-limonene and D-carvone possess strong antifungal activity against several fungi, such as *Aspergillus niger*, *Saccharomyces cerevisiae,* and *Candida albicans* [41,42]. In some studies, it has been shown that *S. aureus* is implicated in alimentary toxic infections [43]. In addition, dill EO can be used as a natural antimicrobial agent in milk products to kill *S. aureus* and its enterotoxins [44]. 

*Salmonella* is Gram-negative bacteria that cause gastroenteritis. The most common sources of food infections are milk, eggs, meat, and meat products [45]. The dill seeds were studied for their antimicrobial activity against *Salmonella* typhimurium [46]. *L. monocytogenes* causes listeriosis after food consumption. Listeriosis can contribute to death in 30% of cases [45]. Many studies focused on the effect of essential oils on *L. monocytogenes* [46]. de Carvalho et al. [47] exhibited the antimicrobial effect of carvone, a major component of dill oil, against *L. monocytogenes*. The high amount of carvone, a compound known for its numerous biological properties, indicates the possible application of dill seed essential oil for medical purposes outside the food industry as a bioactive product of natural origin [48]. There is also data on limonene’s antimicrobial activity [49].

Gene expression is regulated by the mechanisms of bacterial cell communication. It has been demonstrated that the signal molecules of bacteria and the suppression of QS can be affected by some plant compounds [50]. The interaction between plants and bacteria has been reported for many years [50]. During our study, we investigated in vitro the anti-QS potential of *A. graveolens* EO and limonene. This plant is known for its culinary uses as a spice and flavor, as well as its medicinal uses. The results showed that *A. graveolens* EO and limonene inhibited violacein production in *C. violaceum* ATCC 12472, highlighting their ability to inhibit bacterial cell-to-cell communication and consequently interfere with the QS system regulating the production of virulence traits responsible for bacterial disease [51]. Computational studies are commonly used to correlate the in vitro activities of natural compounds with key target proteins involved in human disorders. In fact, in silico docking studies can provide useful insights into the molecular basis of the biological activity of natural products and the possible mechanisms of action and binding modes of active compounds. Therefore, all compounds from the GC-MS analysis of compounds from the tested essential oil were docked with specific target proteins involved in antibacterial and antioxidant activities [52,53]. 

In this target, the co-crystallized ligand benzoic acid has the highest docking score (−7.245 kcal/mol), while docking values ranging from −5.105 to −4.332 kcal/mol were found to have significant binding affinities with dihydrocarvone cis, trans-caranone, and dihydrocarvone (neoiso). The human peroxiredoxin family has one cysteine residue, Cys47, that is shared by all peroxiredoxins and has been attributed to peroxide catalysis. Cys47, a conserved cysteine residue, is located at the N-terminus of the kinked helix 2. This active pocket comprises conserved amino acid residues such as Thr44, Gly46, Cys47, and Arg127 that assist in docked chemical identification through hydrogen bonding and hydrophobic interactions. Furthermore, Cys47, Thr44, Gly46, Thr147, Pro40, Pro45, Phe120, Arg127, and Leu149 are all involved in the complex benzoic acid-1 HD2 stabilization [53]. 

**Figure 9:**
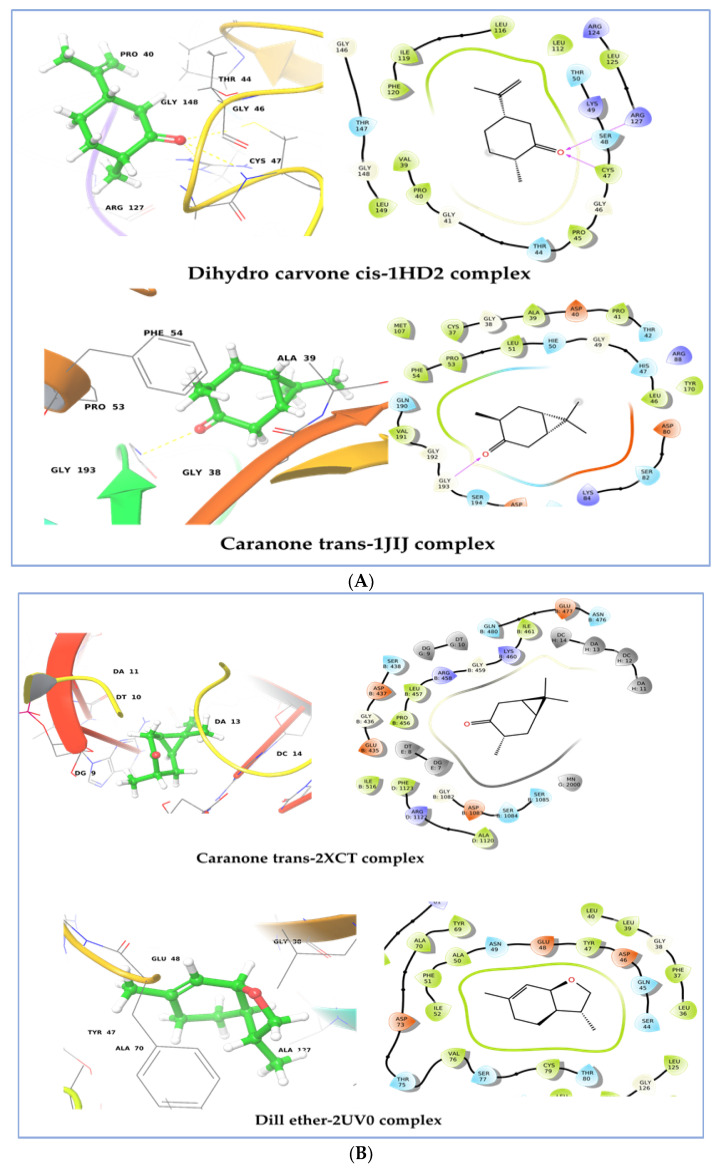

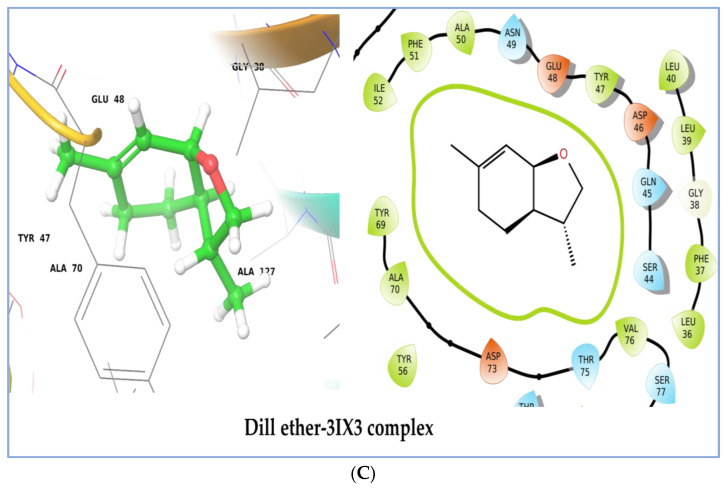
(**A**) 2D and 3D residual interactions network of Dihydro carvone cis-1HD2 complex and Caranone trans-1JIJ complex. (**B**) 2D and 3D residual interaction networks of Caranone trans-2XCT complex and Dill ether-2UV0 complex. (**C**) 2D and 3D residual interaction networks of Dill ether-3IX3 complex.

A closer look at the docking poses of the screened phyto-compounds revealed the presence of a hydrogen bond with Cys47 in dihydrocarvone cis, caranone trans, and dihydrocarveol (neoiso), as well as benzoic acid (Figure 9A), indicating that these three compounds have antioxidant potential.

Antimicrobial medicines often impede cell wall biosynthesis, protein synthesis, nucleic acid synthesis, and anti-metabolism. Antibiotics, in general, disrupt these pathways by interfering with specific cell proteins that perform specific activities [54]. Tyrosyl-tRNA synthetase (TyrRS), a member of the aminoacyl-tRNA synthetase family, can interpret information such as concurrent tRNA molecules and amino acid structures, which are essential for translating coded information into protein structures in nucleic acids. Since this enzyme is highly conserved across prokaryotes, inhibiting it is a promising target for the development of broad-spectrum antibiotics. To better understand the binding interactions, phyto-constituents from dill essential oil compounds were docked in the active site of the crystal structure of *S. aureus* TyrRS (PDB ID: 1JIJ) [55], which was co-crystallized with the monocyclic SB-239629. In *S. aureus* tyrosyl-tRNA synthetase (TyrRS) (1JIJ), the docking scores of phyto-constituents ranged from −3.521 to −5.62 kcal/mol, while the co-crystallized ligand had −7.973 kcal/mol. Molecular docking of caranone trans, dihydro carvone cis, and dihydro carveol (neoiso), the top three compounds that have the best binding affinity, was performed to identify their binding sites on the structures of TyrRS *S. aureus*. In this target, caranone trans has the best docking score, which is −5.622 kcal/mol. The carbonyl group of caranone forms a single hydrogen bond with the amino acid Gly193. The compound trans-caranone interacted with Ser194, Gly192, Val191, Gln190, Asp195, Leu46, His47, and Asp80 at the binding site via van der Waals bonding (Figure 9B).

DNA gyrase is a bacterial topoisomerase enzyme that controls the structure of DNA during transcription, replication, and recombination by generating transitory breaks in both DNA strands [53]. As a result, this enzyme is critical for bacterial survival and may primarily be used as an antibacterial targeted therapy. The highest scoring ligands in a docking study on topoisomerase II DNA gyrase (PDB ID, 2XCT) protein were ρ-cymene (−6.204 kcal/mol), meta-cymene (−6.238 kcal/mol), dill ether (−6.282 kcal/mol), and trans-caranone (−6.326 kcal/mol). The binding interaction of the most active ligand, trans-caranone, shows that there is no direct hydrogen bonding between the ligand atom and protein residues. In fact, trans-caranone is situated on the two DNA helices and makes van der Waals interactions with nucleotide bases such as DT E8, DT E9, DC H14, DA H13, DC H12, and DA H11. Trans-caranone binding contact was unable to exhibit interaction with the Mn^+2^ ion through a salt bridge, which dramatically increased rates of enzyme-mediated DNA breaking reported in previous research (Figure 9C).

In order to control the actions of their populations, many Gram-negative bacteria communicate through chemicals known as autoinducers. Quorum sensing is a kind of communication that may control the production of virulence factors, biofilm formation, and drug susceptibility [56,57]. The quorum sensing system in the human opportunistic bacterium *P. aeruginosa* is now the most extensively studied one. *P. aeruginosa* pathogenicity may be reduced by using quorum sensing inhibitors. For the quorum sensing inhibition activity, docking studies on the LasR protein receptor of *P. aeruginosa* (PDB ID: 2UV0 and 3IX3) were performed. Dill ether was chosen as the best natural ligand against 2UV0 and 3IX3 thanks to their good binding affinity within the active site domain, which is −6.976 kcal/mol and −7.077 kcal/mol, respectively, which is comparable to a co-crystallized ligand. Binding interaction shows that dill ether forms van der Waals interactions with Ser40, Gln45, Asp46, Tyr47, Glu48, Asn49, Ala50, Phe51, Ile52, Val76, Ser77, Cys79, and Thr80 in the *P. aeruginosa* 2UV0 protein target, which is a comparable binding site for co-crystallized ligand.

In this target co-crystalized ligand, acyl homoserine lactone is housed in a large hydrophobic pocket formed by residues Leu36, Gly38, Leu39, Leu40, Tyr47, Glu48, Ala50, Ile52, Tyr56, Trp60, Arg61, Tyr64, Asp65, Gly68, Tyr69, Ala70, Asp73, Pro74, Thr75, Val76, Cys79, Thr80, Trp88, Tyr93, Phe101, Phe102, Ala105, Leu110, Thr115, Leu125, Gly126, Ala127, and Ser129. While in 3IX3 protein, Dill ether is situated in a cage of hydrophobic and acidic amino acids, such as Tyr69, Ala70, Asp73, Thr75, Val76, Ser44, Gln45, Asp46, Tyr47, Glu48, Asn49, Ala50, Phe51, and Ile52 [56]. 

The presence of dill ether in the LasR receptor’s hydrophobic pocket indicated the existence of hydrophobic interactions created by the benzofuran scaffold and hydrophobic amino acids. Thus, molecular docking studies have shown the ability of the dill ether structural structure to inhibit the quorum sensing mechanism.

## 4. Materials and Methods

### 4.1. Chemical Composition Analyses

*Anethum graveolens* essential oil and its major compound limonene were purchased from Huile & Sens (Crestet, France) and Sigma (Sigma-Aldrich S.r.l., Milan, Italy), respectively. The chemical composition of the essential oil was analyzed by gas chromatography–flame ionization detector (GC–FID) and gas chromatography–mass spectrometry (GC–MS) [58].

### 4.2. Disk Diffusion Test 

The antagonistic effects of *A. graveolens* EO and limonene were evaluated against eight pathogenic bacterial strains: *Listeria monocytogenes* CECT 933; *Vibrio vulnificus* CECT 529; *Salmonella enterica* CECT 443; *Shigella flexeneri* CECT 4804; *Staphylococcus aureus* ATCC 6538; *Bacillus subtilis* CIP 5265; *Escherichia coli* ATCC 35218; and *Pseudomonas aeruginosa* PAO1. The disk diffusion method was performed according to Pérez et al. [59]. The results were determined as the diameters of inhibition zones (mm) around discs impregnated with EO. Gentamicin discs were taken as positive controls.

### 4.3. Microdilution Assay

The minimal inhibition concentration (MIC) and the minimal bactericidal concentration (MBC) of *A. graveolens* EO and its major compound were determined for all bacterial strains as previously described [60]. Serial dilution of the tested agents was performed at concentrations ranging from 50 to 0.048 mg/mL.

### 4.4. Adhesive Potentiality 

The ability of the tested microorganisms to secrete exopolysaccharides on Congo red agar plates was determined, and morphotypes obtained were defined on the basis of their color (slime production) using the protocol described by Touati et al. [61]. Qualitative adhesion on glass tubes was carried out following the same protocol described by Davenport et al. [62]. All experiments were done in triplicate. 

Quantitative biofilm formation on a polystyrene 96-well plate was determined using the crystal violet technique [63]. 

### 4.5. Biofilm Formation Capacity on Abiotic Surfaces

Polyvinyl chloride (PVC), stainsteel (SS), and glass (G) strips (1.5 cm^2^) were disinfected before being used for the biofilm assay. A volume of 100 µL of bacterial suspensions was added to each strip placed into a 12-well tissue culture plate. After incubation (24 h at 37 °C), non-adherent cells were removed from each well by washing with PBS solution. Biofilm quantification was made with crystal violet (1%) staining and then dissolved into acetic acid (33%). The OD at 570 nm was recorded [64].

### 4.6. Anti-Biofilm Activities 

#### 4.6.1. Biofilm Inhibition 

The biofilm inhibition effects of *A. graveolens* EO and limonene were evaluated according to the protocol of Saising et al. [65]. Each bacterial strain grown in BHI (with 2% glucose) was treated with different subinhibitory concentrations (1/16 to 1 × MIC) of the tested agents. After incubation for 24 h at 37 °C, non-adherent cells were removed, and CV (1%) stained biofilm cells were determined at 570 nm. 

#### 4.6.2. Biofilm Eradication

The biofilm eradication properties of *A. graveolens* EO and limonene were tested as described previously [66]. Pre-established biofilms (48 h) were treated with various concentrations ranging from MIC to 4 × MIC of the selected agents and further incubated for 24 h. Treated biofilm biomass was stained with CV (1%) and measured by the absorbance of CV at 570 nm. The percentage of biofilm eradication was estimated using the following equation (Equation (1)):

[(OD growth control − OD sample)/OD growth control] × 100(1)

### 4.7. Anti-Quorum Sensing Activity

The ability of *A. graveolens* EO and limonene to inhibit the production of the water-soluble pigment (violacein) in the *Chromobacterium violaceum* ATCC 12472 starter strain was evaluated. An overnight culture of *C. violaceum* (OD_600_ = 0.4) was added to sterile microtiter plates containing 200 μL of LB broth and incubated at 30 °C supplemented with different concentrations (MIC/32 to MIC) of dill EO and limonene. LB broth containing *C. violaceum* was used as a positive control [67]. The percentage of violacein reduction was calculated by the following equation (Equation (2)):

Violacein inhibition (%) = (OD_585_ Control − OD_585_ Sample)/OD_585_ Control (2)

### 4.8. Anti-Swarming Activity

Anti-swarming activity of *A. graveolens* EO and limonene was assessed against *Pseudomonas aeruginosa* PAO1. An overnight culture of PAO1 (OD600 = 0.4) was inoculated on the swarming agar medium at various concentrations of test agents (50, 75, and 100 μg/mL). Plates without EOs were considered controls. After incubation for 18 h at 30 °C [68]. 

### 4.9. ADMET Profile

The pharmacokinetics and the toxicity profiles of the identified molecules were predicted using a SwissADME online server (http://www.swissadme.ch/, accessed on 19 January 2022) and a ProTox-II webserver (http://tox.charite.de/tox/, accessed on 19 January 2022) [69,70,71].

### 4.10. Molecular Docking Study

In order to highlight the possibility of binding interactions between phytocompounds identified in *A. graveolens* essential oil and antimicrobial, antioxidant, and quorum sensing receptors, a docking approach was performed. For antimicrobial activity, *S. aureus* tyrosyl-tRNA synthetase (PDB ID, 1JIJ) and topoisomerase II DNA gyrase (PDB ID, 2XCT) proteins are promising drug candidates, leading to high selectivity and a broad spectrum of antibacterial agents [72,73]. The human peroxiredoxin 5 (PRDX5) receptor (PDB ID, 1HD2) is a potential target for the evaluation of the antioxidant activity of selected bioactive compounds that permit the reduction of hydrogen peroxide and alkyl peroxide with the help of thiol-containing donor molecules [1,2]. A molecular docking study was also performed against the QS signal receptors LasR (PDB ID: 2UV0) and (PDB ID: 3IX3), from *P. aeruginosa* as key regulators of *P. aeruginosa* pathogenesis.

The phyto-constituents 2D structures were obtained using PubChem chemical information resources. The LigPrep module was used to refine the structure obtained. The OPLS3e force field was applied for ligand preparation. Tautomer creation, ionization state at pH 7.0 ± 1.0 utilizing Epik, charged group neutralization, and optimization of the hydrogen bond and ligand 3D geometry are all included in the ligand preparation process [74,75,76]. Protein PDB IDs 1HD2, 1JIJ, 2XCT, 2UV0, and 3IX3 are downloaded by the protein data bank and processed for the modeling study. The protein was imported into the Schrodinger Maestro GUI and refined, optimized, and minimized after undesired water molecules and problem warnings were removed using the protein preparation wizard module [77,78]. The Prime tool was applied to complete the missing side chains and residues. The OPLS3e force field was utilized to construct low-energy state proteins with a default root-mean-square deviation (RMSD) of 0.30 Å, which were then employed for molecular docking. 

The grid is constructed from minimized proteins, and the grid box was constructed based on the active site of the protein where the co-crystallized ligand is bound [79,80]. Glide was used to undertake molecular docking simulations using the standard precision (SP) approach, which also produced favorable ligand poses for further evaluating active sites for ligand binding. The docking results included the best positions as well as the dock score.

### 4.11. Statistical Analysis

All experiments were performed in triplicate, and average values were calculated using the SPSS 25.0 statistical package for Windows. Differences in means were calculated using Duncan’s multiple range tests for means with a 95% confidence interval (*p* ≤ 0.05). For anticancer activities, a significance test was carried out among the treatments by a two-way ANOVA followed by a Bonferroni post hoc test at *p* < 0.001.

## 5. Conclusions

*Anethum graveolens* is a very rich plant in phytochemicals with a pharmacological interest. Docking studies on the identified phyto-compounds in bacteria were studied to reinforce the in vitro results. The results obtained confirm the alternative use of this plant to treat human diseases because of its effectiveness and safety. *A. graveolens* EO is a considerable natural antibacterial agent and might be used as a natural preservative in the food industry.

## Figures and Tables

**Table 1:** Phytochemical composition of *A. graveolens* EO by using GC–EIMS technique.

N.	Compound	Percentage	Ki ^a^	Ki ^b^
1	α-pinene	0.68	924	939
2	β-pinene	0.22	984	979
3	δ-2-carene	0.23	995	1002
4	ρ- cymene	0.26	1016	1026
5	Limonene	48.05	1017	1029
6	Meta-cymene	0.10	1082	1037
7	Limonene oxide	0.26	1039	1050
8	Dill ether	0.11	1179	1059
9	Cis-Dihydro carvone	3.5	1190	1070
10	Caranone trans	1.07	1197	1072
11	Dihydro carveol (neoiso)	0.13	1227	1090
12	Carvone	37.94	1244	1096
Total (%)		92.55		

^a^ Kovats retention index determined relative to the tR of a series of n-alkanes (C10–C35) on a HP-5 MS column; ^b^ Kovats retention index determined relative to the tR of a series of n-alkanes (C10–C35) on HP Innowax.

**Table 2:** Antibacterial activity of *A. graveolens* EO and limonene against pathogenic bacteria evaluated recorded as inhibition zones, MICs, and MBCs values.

Strains	*A. graveolens* EO			MBC/MIC Ratio	Limonene			MBC/MIC Ratio
	IZ ± SD (mm)	MIC (mg/mL)	MBC (mg/mL)		IZ ± SD (mm)	MIC (mg/mL)	MBC (mg/mL)	
*Listeria monocytogenes* CECT 933	28.5 ± 0.71	0.048	>50	>4; Bacteriostatic	11.66 ± 0.57	0.048	50	>4; Bacteriostatic
*Vibrio vulnificus* CECT 529	22.5 ± 0.71	0.048	>50	>4; Bacteriostatic	6 ± 0.1	0.048	>50	>4; Bacteriostatic
*Shigella flexeneri* CECT 4804	21.66 ± 0.57	0.097	12.5	>4; Bacteriostatic	8 ± 0.1	0.048	>50	>4; Bacteriostatic
*Bacillus subtilis* CIP 5265	27.66 ± 0.57	0.048	50	>4; Bacteriostatic	6 ± 0.1	0.048	50	>4; Bacteriostatic
*Salmonella enterica* CECT 443	20 ± 1	0.048	>50	>4; Bacteriostatic	17 ± 0.81	0.048	>50	>4; Bacteriostatic
*Escherichia coli* ATCC 35218	10 ± 0.1	0.048	>50	>4; Bacteriostatic	7 ± 0.1	0.048	50	>4; Bacteriostatic
*Pseudomonas aeruginosa* PAO1	6 ± 0.1	0.048	12.5	>4; Bacteriostatic	6 ± 0.1	0.048	>50	>4; Bacteriostatic
*Staphylococcus aureus* ATCC 6538	21 ± 1	0.048	50	>4; Bacteriostatic	10.33 ± 0.57	0.048	>50	>4; Bacteriostatic

IZ: inhibition zone; SD: standard deviation; MIC: minimum inhibitory concentration; MBC: minimal bactericidal concentration.

**Table 3:** Adhesive properties of selected pathogenic strains.

Strains	Adhesion to Glass	Exopolysaccharide Production on CRA		Adhesion to Polystyrene	
		Colour	S+/S−	OD_570_ ± SD	Biofilm Production
*S. aureus*	++	Black	S+	1.36 ± 0.2	High producer
*L. monocytogenes*	+++	Red with black center	S+	0.19 ± 0.07	Moderate producer
*V. vulnificus*	++	Red with black center	S+	0.13 ± 0.02	Moderate producer
*B. subtilis*	+	Red bordeaux	S−	0.12 ± 0.01	Moderate producer
*E. coli*	++	Red with black center	S+	0.17 ± 0.03	Moderate producer
*S. flexeneri*	+++	Red with black center	S+	0.10 ± 0.01	Moderate producer
*S. enterica*	+	Red bordeaux	S−	0.15 ± 0.01	Moderate producer
*P. aeruginosa*	++	Red bordeaux	S−	0.42 ± 0.26	Moderate producer

CRA: Congo red agar; S+: slime-positive: S−: slime-negative; OD: optical density; SD: standard deviation; Weak slime production (+), moderate slime production (++), or strong slime production (+++).

**Table 4:** Effect of *A. graveolens* EO and limonene on swarming motility of PAO1.

EO/Main Compound	Concentration	% Anti-Swarming Activity
*A. graveolens*	50 µg/mL	16.67 ± 0
	75 µg/mL	20.83 ± 1.17
	100 µg/mL	33.33 ± 0
Limonene	50 µg/mL	10.4 ± 1.3
	75 µg/mL	19.22 ± 2.1
	100 µg/mL	28.9 ± 0.9

**Table 5:** Qualitative violacein inhibition on *C. violaceum* ATCC 12472.

Concentration	% of Violacein Inhibition	
	*A. graveolens*	Limonene
MIC	67.52 ± 1.1	18.68 ± 1.6
MIC/2	57.91 ± 1.8	10.53 ± 1.1
MIC/4	46.77 ± 2.3	9.12 ± 2.3
MIC/8	33.56 ± 1	6.33 ± 1
MIC/16	29.93 ± 1.7	1.33 ± 1.3
MIC/32	23.66 ± 1.6	1.03 ± 0.9

MIC: Minimum inhibitory concentration; MIC *A. graveolens*: 10 mg/mL; MIC limonene: 1.25 mg/mL.

**Table 6:** Selected ADME properties of identified compounds in *A. graveolens* EO. Number and name of the compounds are the same as listed in Table 1.

Entry	1	2	3	4	5	6	7	8	9	10	11	12
Physicochemical Properties												
Molecular weight (g/mol)	136.23	136.23	136.23	136.23	136.23	132.20	152.23	152.23	152.23	168.23	154.25	150.22
Num. heavy atoms	10	10	10	10	10	10	11	11	11	12	11	11
Num. arom. heavy atoms	0	0	0	0	0	6	0	0	0	0	0	0
Fraction Csp3	0.80	0.80	0.80	0.80	0.60	0.20	0.80	0.80	0.70	0.90	0.80	0.50
Num. rotatable bonds	0	0	0	0	1	1	1	0	1	0	1	1
Num. H-bond acceptors	0	0	0	0	0	0	1	1	1	2	1	1
Num. H-bond donors	0	0	0	0	0	0	0	0	0	1	1	0
Molar Refractivity	45.22	45.22	45.22	45.22	47.12	46.31	46.60	46.57	47.80	47.10	48.76	47.32
TPSA (Å²)	0.00	0.00	0.00	0.00	0.00	0.00	12.53	9.23	17.07	37.30	20.23	17.07
Consensus Log Po/w	3.44	3.42	3.12	3.5	3.37	3.37	2.71	2.27	2.51	1.56	2.48	2.44
Lipinski rules	Yes	Yes	Yes	Yes	Yes	Yes	Yes	Yes	Yes	Yes	Yes	Yes
Bioavailability Score	0.55	0.55	0.55	0.55	0.55	0.55	0.55	0.55	0.55	0.55	0.55	0.55
Pharmacokinetics												
GI absorption	Low	Low	Low	Low	Low	Low	High	High	High	High	High	High
BBB permeant	Yes	Yes	Yes	Yes	Yes	Yes	Yes	Yes	Yes	Yes	Yes	Yes
P-gp substrate	No	No	No	No	No	No	No	No	No	No	No	No
CYP1A2 inhibitor	No	No	No	No	No	No	No	No	No	No	No	No
CYP2C19 inhibitor	No	No	No	No	No	No	No	No	No	No	No	No
CYP2C9 inhibitor	Yes	Yes	No	No	Yes	No	No	No	No	No	No	No
CYP2D6 inhibitor	No	No	No	Yes	No	Yes	No	No	No	No	No	No
CYP3A4 inhibitor	No	No	No	No	No	No	No	No	No	No	No	No
Log *K*_p_ (cm/s)	−3.95	−4.18	−5.11	−4.21	−3.89	−4.49	−4. 6	−5.88	−5.21	−6.41	−4.96	−5.29

**Table 7:** Docking score of identified phyto-constituents with the antioxidant, antibacterial, and anti-QS activities targets.

No.	Compound	1HD2	1JIJ	2XCT	2UV0	3IX3
1	α-pinene	−3.86	−4.526	−5.707	−6.614	−6.585
2	β-pinene	−3.597	−4.499	−5.512	−6.574	−6.546
3	δ-2-carene	−3.86	−4.526	−5.707	−6.614	−6.585
4	ρ-cymene	−3.336	−4.481	−6.204	−6.727	−6.774
5	Limonene	−3.032	−3.521	−5.525	−6.153	−5.874
6	Meta-cymene	−3.537	−4.592	−6.238	−6.875	−7.051
7	Limonene oxide	−3.309	−4.323	−5.525	−6.547	−5.917
8	Dill ether	−3.99	−4.716	−6.282	−6.976	−7.077
9	Cis-Dihydro carvone	−5.105	−4.826	−5.769	−6.401	−6.267
10	Trans-caranone	−4.642	−5.622	−6.326	−6.806	−6.958
11	Dihydro carveol (neoiso)	−4.332	−4.916	−5.769	−6.771	−6.739
12	Carvone	−3.529	−4.894	−5.621	−5.529	−5.964
	Co-crystal inhibitor	−7.245	−7.973	−8.521	−6.929	−6.057

## Data Availability

Not applicable.

## References

[1] Vaou N., Stavropoulou E., Voidarou C., Tsigalou C., Bezirtzoglou E. (2021). Towards Advances in Medicinal Plant Antimicrobial Activity: A Review Study on Challenges and Future Perspectives. Microorganisms.

[2] Aminzare M., Hashemi M., Azar H.H., Hejazi J. (2016). The use of herbal extracts and essential oils as a potential antimicrobial in meat and meat products; a review. J. Hum. Environ. Health Promot..

[3] Al-Haidari R.A., Shaaban M.I., Ibrahim S.R.M., Mohamed G.A. (2016). Anti-quorum sensing activity of some medicinal plants. Afr. J. Tradit. Complement. Altern. Med..

[4] Jamal M., Ahmad W., Andleeb S., Jalil F., Imran M., Nawaz M.A., Hussain T., Ali M., Rafiq M., Kamil M.A. (2018). Bacterial biofilm and associated infections. J. Chin. Med. Assoc..

[5] Mseddi K., Alimi F., Noumi E., Veettil V.N., Deshpande S., Adnan M., Hamdi A., Elkahoui S., Alghamdi A., Kadri A. (2020). Thymus musilii Velen. as a promising source of potent bioactive compounds with its pharmacological properties: In vitro and in silico analysis. Arab. J. Chem..

[6] Chopra I., Hodgson J., Metcalf B., Poste G. (1997). The search for antibacterial agents effective against bacteria resistant to multiple antibiotics. Antimicrob. Agents Chemother..

[7] Rojas J.J., Ochoa V.J., Ocampo S.A., Munoz J.F. (2006). Screening for antimicrobial activity of ten medicinal plants used in Colombian folkloric medicine: A possible alternative in the treatment of non-nosocomial infections. BMC Complement Altern. Med..

[8] Shan B., Cai Y.Z., Brooks J.D., Corke H. (2007). The in vitro antibacterial activity of dietary spice and medicinal herb extracts. Int. J. Food Microbiol..

[9] Tepe B., Daferera D., Sokmen M., Polissiou M., Sokmen A. (2004). In vitro antimicrobial and antioxidant activities of the essential oils and various extracts of Thymus eigii M. Zohary et P.H. Davis. J. Agric. Food Chem..

[10] Wren R.C., Walden S. (1988). Potter’s New Cyclopedia of Botanical Drugs and Preparations.

[11] Leung A.Y., Foster S. (1996). Encyclopedia of Common Natural Ingredients Used in Food, Drugs and Cosmetics.

[12] Yazdanparast R., Bahramikia S. (2008). Evaluation of the effect of *Anethum graveolens* L. crude extracts on serum lipids and lipoproteins profiles in hypercholesterolaemic rats. DARU J. Pharm. Sci..

[13] Lagos, Organization of African Unity, Scientific Technical and Research Commission (1985). African Pharmacopoeia.

[14] Zargari A. (1996). Medicinal Plants.

[15] Ishikawa T.M., Kudo M., Kitajima J. (2002). Water-soluble constituents of dill. Chem. Pharm. Bull..

[16] Kaur G.J., Arora D.S. (2010). Bioactive potential of *Anethum graveolens, Foeniculum vulgare* and *Trachyspermum ammi* belonging to the family Umbelliferae-Current status. J. Med. Plants Res..

[17] Leung A.Y., Foster S. (2003). Encyclopedia of Common Natural Ingredients (Used in Food, Drugs, and Cosmetics).

[18] Hosseinzadeh H., Karimi G.R., Ameri M. (2002). Effects of *Anethum graveolens* L. Seed extracts on experimental gastric irritation models in mice. BMC Pharmacol..

[19] Vishaldeep K., Ramandeep K., Urvashi B. (2021). A review on dill essential oil and its chief compounds as natural biocide. Flavour Fragr. J..

[20] Rădulescu V., Popescu M.L., Ilieş D.C. (2010). Chemical composition of the volatile oil from different plant parts of *Anethum graveolens* L. (*Umbelliferae*) cultivated in Romania. Farmacia.

[21] Stanojević L.P., Radulović N.S., Djokić T.M., Stanković B.M., Ilić D.P., Cakić M.D., Nikolić V.D. (2015). The yield, composition and hydrodistillation kinetics of the essential oil of dill seeds (*Anethii fructus*) obtained by different hydrodistillation techniques. Indus. Crops Prod..

[22] Jump up- Saiman L. (2004). Microbiology of early CF lung disease. Paedia. Resp. Rev..

[23] Dhama k., Tiwari R., Chakraborty S., Saminathan M., Kumar A., Karthik K., Wani Y., Amarpal S.V.S., Rahal A. (2014). Evidence Based Antibacterial Potentials of Medicinal Plants and Herbs Countering Bacterial Pathogens Especially in the Era of Emerging Drug Resistance: An Integrated Update. Int. J. Pharmacol..

[24] Meng X.Y., Zhang H.X., Mezei M., Cui M. (2011). Molecular docking: A powerful approach for structure-based drug discovery. Curr. Comput. Aided Drug Des..

[25] Jorgensen W.L. (2004). The many roles of computation in drug discovery. Science.

[26] Bajorath J. (2002). Integration of virtual and high-throughput screening. Nat. Rev. Drug Discov..

[27] Haddaji F., Papetti A., Noumi E., Colombo R., Deshpande S., Aouadi K., Adnan M., Kadri A., Selmi B., Snoussi M. (2021). Bioactivities and in silico study of *Pergularia tomentosa* L. phytochemicals as potent antimicrobial agents targeting type IIA topoisomerase, TyrRS, and Sap1 virulence proteins. Environ. Sci. Pollut. Res. Int..

[28] Noumi E., Snoussi M., Anouar E.H., Alreshidi M., Veettil V.N., Elkahoui S., Adnan M., Patel M., Kadri A., Aouadi K. (2020). HR-LCMS-based metabolite profiling, antioxidant, and anticancer properties of *Teucrium polium* L. methanolic extract: Computational and in vitro study. Antioxidants.

[29] Badraoui R., Saeed M., Bouali N., Hamadou W.S., Elkahoui S., Alam M.J., Siddiqui A.J., Adnan M., Saoudi M., Rebai T. (2022). Expression Profiling of Selected Immune Genes and Trabecular Microarchitecture in Breast Cancer Skeletal Metastases Model: Effect of α-Tocopherol Acetate Supplementation. Calcif. Tissue Int..

[30] Gatsing D., Tchakoute V., Ngamga D., Kuiate J.R., Tamokou J.D.D., Nji-Nkah B.F., Tchouanguep F.M., Fodouop S.P.C. (2009). In vitro antibacterial activity of Crinum purpurascens Herb. leaf extract against the Salmonella species causing typhoid fever and its toxicological evaluation. Iran J. Med. Sci..

[31] Moroh J.L., Bahi C., Dje K., Loukou Y.G., Guide Guina F. (2008). Etude de l’activité antibactérienne de l’extrait acétatique de Morinda morindoides (Baker) Milne-Redheat (*Rubiaceae*) sur la croissance in vitro des souches d’Escherichia coli. Bull. Soc. R Sci. Liege.

[32] Al-Ma’adhedi S.H.F. (2012). Phytochemical Screening, Estimation of Some Heavy Metals Concentrations, and Specific Extraction of Bioactive Components from Iraqi *Anethum graveolens* L. Seeds and Studying their Antibacterial Activity. Anbar J. Vet. Sci..

[33] Singh G., Maurya S., de Lampasona C.C. (2006). Chemical Constituents, Antimicrobial Investigations, and Antioxidative Potentials of *Anethum graveolens* L. Essential Oil and Acetone Extract. J. Food Sci..

[34] Singh G., Kapoor I.P.S., Pandey S.K. (2001). Studies on essential oils. Antibacterial activity of volatile oils of some spices. Phytother. Res..

[35] Rifatuz-Zaman M.S., Akhtar M.S., Khan M.S. (2006). In vitro antibacterial screening of *Anethum graveolens* L. Fruit, *Cichorium intybus* L. leaf, Plantago ovata L. seed husk and *Polygonum viviparum* L. root extracts against *Helicobacter pylori*. Int. J. Pharmacol..

[36] Jana S., Shekhawat G.S. (2010). Phytochemical Analysis and Antibacterial Screening of in vivo and in vitro Extracts of Indian Medicinal Herb: *Anethum graveolens*. Res. J. Med. Plant..

[37] Johnson S. (2014). How to Distill Peppermint Oil. J. Ess. Oil. Bear. Plants.

[38] Viuda-Martos M., Ruíz-Navajas Y., Fernández-López J., Pérez-Álvarez J.A. (2007). Chemical composition of the essential oils obtained from some spices widely used in Mediterranean region. Acta Chim. Slov..

[39] Karpiński T.M. (2020). Essential Oils of Lamiaceae Family Plants as Antifungals. Biomolecules.

[40] Jianu C., Misca C., Pop G., Rusu L.C., Ardelean L., Gruia A.T. (2012). Chemical composition and antimicrobial activity of essential oils obtained from dill (*Anethum graveolens* L.) grown in western Romania. Rev. Chim..

[41] Kaur G.J., Arora D.S. (2008). In vitro antibacterial activity of three plants belonging to the family *Umbelliferae*. Int. J. Antimicrob. Agents..

[42] Kaur G.J., Arora D.S. (2009). Antibacterial and phytochemical screening of *Anethum graveolens, Foeniculum vulgare* and *Trachyspermum ammi*. BMC Complement. Altern. Med..

[43] Milanov D., Lazić S., Vidić B., Petronijević J., Bugarski D., Šugeljev Z. (2010). Slime production and biofilm forming ability by *Staphylococcus aureus* bovine mastitis isolates. Acta Vet..

[44] Samaržija D., Damjanović S., Pogačić T. (2007). *Staphylococcus aureus* u siru. Mljekarstvo.

[45] Markov K., Frece J., Čvek D., Delaš F. (2009). Listeria monocytogenes i drugi kontaminanti u svježem siru i vrhnju domaće proizvodnje s područja grada Zagreba. Mljekarstvo.

[46] Delaquis P.J., Stanich K., Girard B., Mazza G. (2002). Antimicrobial activity of individual and mixed fractions of dill, cilantro, coriander and eucalyptus essential oils. Int. J. Food Microbiol..

[47] de Carvalho C.C.C.R., da Fonseca M.M.R. (2006). Carvone: Why and how should one bother to produce this terpene. Food Chem..

[48] Bakkali F., Averbeck S., Averbeck D., Idaomar M. (2008). Biological effects of essential oils-A review. Food Chem. Toxicol..

[49] Saleh-E-In M.M., Sultana N., Rahim M.M., Ahsan M.A., Bhuiyan M.N., Hossain M.N., Rahman M.M., Kumar Roy S., Islam M.R. (2017). Chemical composition and pharmacological significance of *Anethum* Sowa L. Root. BMC Complement Altern Med..

[50] Bauer W.D., Teplitski M. (2001). Can plants manipulate bacterial quorum sensing?. Aust. J. Plant Physiol..

[51] Mohammadi Pelarti S., Karimi Zarehshuran L., Babaeekhou L., Ghane M. (2021). Antibacterial, anti-biofilm and anti-quorum sensing activities of Artemisia dracunculus essential oil (EO): A study against Salmonella enterica serovar Typhimurium and *Staphylococcus aureus*. Arch. Microbiol..

[52] Zala A.R., Dhanji P.R., Iqrar A., Haruny P., Premlata K. (2023). Synthesis, characterization, molecular dynamic simulation, and biological assessment of cinnamates linked to imidazole/benzimidazole as a CYP51 inhibitor. J. Biomol. Struct. Dyn..

[53] Noumi E., Ahmad I., Bouali N., Patel H., Ghannay S., ALrashidi A.A., Snoussi M. (2023). *Thymus musilii* Velen. Methanolic Extract: In Vitro and In Silico Screening of Its Antimicrobial, Antioxidant, Anti-Quorum Sensing, Antibiofilm, and Anticancer Activities. Life.

[54] Alsagaby S.A., Iqbal D., Ahmad I., Patel H., Mir S.A., Madkhali Y.A., Oyouni A., Hawsawi Y.M., Alhumaydhi F.A., Alshehri B. (2022). In silico investigations identified Butyl Xanalterate to competently target CK2α (CSNK2A1) for therapy of chronic lymphocytic leukemia. Sci. Rep..

[55] Snoussi M., Ahmad I., Aljohani A.M.A., Patel H., Abdulhakeem M.A., Alhazmi Y.S., Tepe B., Adnan M., Siddiqui A.J., Sarikurkcu C. (2022). Phytochemical Analysis, Antioxidant, and Antimicrobial Activities of *Ducrosia flabellifolia*: A Combined Experimental and Computational Approaches. Antioxidants.

[56] Bottomley M.J., Muraglia E., Bazzo R., Carfì A. (2007). Molecular insights into quorum sensing in the human pathogen Pseudomonas aeruginosa from the structure of the virulence regulator LasR bound to its autoinducer. J. Biol. Chem..

[57] McCready A.R., Paczkowski J.E., Henke B.R., Bassler B.L. (2019). Structural determinants driving homoserine lactone ligand selection in the Pseudomonas aeruginosa LasR quorum-sensing receptor. Proc. Natl. Acad. Sci. USA.

[58] Noumi E., Merghni A., Alreshidi M., Haddad O., Akmadar G., De Martino L., Mastouri M., Ceylan O., Snoussi M., Al-Sieni A. (2018). *Chromobacterium violaceum* and *Pseudomonas aeruginosa* PAO1: Models for Evaluating Anti-Quorum Sensing Activity of *Melaleuca alternifolia* Essential Oil and Its Main Component Terpinen-4-ol. Molecules.

[59] Perez C., Agnese A.M., Cabrera J.L. (1999). The essential oil of *Senecio graveolens* (*Compositae*): Chemical composition and antimicrobial activity tests. J. Ethnopharmacol..

[60] ALrashidi A.A., Noumi E., Snoussi M., Feo V. (2022). Chemical Composition, Antibacterial and Anti-Quorum Sensing Activities of *Pimenta dioica* L. Essential Oil and Its Major Compound (Eugenol) against Foodborne Pathogenic Bacteria. Plants.

[61] Touati A., Achour W., Abbassi M.S., Ben Hassen A. (2007). Detection of *ica* genes and slime production in a collection of *Staphylococcus epidermidis* strains from catheter-related infections in neutropenic patients. Pathol. Biol..

[62] Davenport D.S., Massanari R.M., Pfaller M.A., Bale M.J., Streed S.A., Hierholzer W.J. (1986). Usefulness of a test for slime production as a marker for clinically significant infections with coagulase-negative staphylococci. J. Infect. Dis..

[63] Mack D., Bartscht K., Fischer C., Rohde H., de Grahl C., Dobinsky S. (2001). Genetic and biochemical analysis of *Staphylococcus epidermidis* biofilm accumulation. Methods Enzymol..

[64] Merghni A., Noumi E., Hadded O., Dridi N., Panwar H., Ceylan O., Mastouri M., Snoussi M. (2018). Assessment of the antibiofilm and antiquorum sensing activities of *Eucalyptus globulus* essential oil and its main component 1,8-cineole against methicillin-resistant *Staphylococcus aureus* strains. Microb. Pathog..

[65] Saising J., Dube L., Ziebandt A.K., Voravuthikunchai S.P., Nega M., Gotz F. (2012). Activity of Gallidermin on *Staphylococcus aureus* and *Staphylococcus epidermidis* biofilms. Antimicrob. Agents Chemother..

[66] Qais F.A., Ahmad I. (2022). Anti-quorum sensing and biofilm inhibitory effect of some medicinal plants against gram-negative bacterial pathogens: In vitro and in silico investigations. Heliyon.

[67] Weiser J., Henke H.A., Hector N., Both A., Christner M., Büttner H., Kaplan J.B., Rohde H. (2016). Sub-inhibitory tigecycline concentrations induce extracellular matrix binding protein Embp dependent Staphylococcus epidermidis biofilm formation and immune evasion. Int. J. Med. Microbiol..

[68] Packiavathy I.A., Priya S., Pandian S.K., Ravi A.V. (2014). Inhibition of biofilm development of uropathogens by curcumin–An anti-quorum sensing agent from Curcuma longa. Food Chem..

[69] Kadri A., Aouadi K. (2020). In vitro antimicrobial and α-glucosidase inhibitory potential of enantiopure cycloalkylglycine derivatives: Insights into their in silico pharmacokinetic, druglikeness, and medicinal chemistry properties. J. App. Pharm. Sci..

[70] Othman I.M.M., Gad-Elkareem M.A.M., Anouar E.H., Aouadi K., Kadri A., Snoussi M. (2020). Design, synthesis ADMET and molecular docking of new imidazo [4,5-b] pyridine-5-thione derivatives as potential tyrosyl-tRNA synthetase inhibitors. Bioorg. Chem..

[71] Othman I.M., Gad-Elkareem M.A., Snoussi M., Aouadi K., Kadri A. (2020). Novel fused pyridine derivatives containing pyrimidine moiety as prospective tyrosyl-tRNA synthetase inhibitors: Design, synthesis, pharmacokinetics and molecular docking studies. J. Mol. Struct..

[72] Ghannay S., Aouadi K., Kadri A., Snoussi M. (2022). GC-MS Profiling, Vibriocidal, Antioxidant, Antibiofilm, and Anti-Quorum Sensing Properties of *Carum carvi* L. Essential Oil: In Vitro and In Silico Approaches. Plants.

[73] Ghannay S., Aouadi K., Kadri A., Snoussi M. (2022). In Vitro and In Silico Screening of Anti-*Vibrio* spp., Antibiofilm, Antioxidant and Anti-Quorum Sensing Activities of *Cuminum cyminum* L. Volatile Oil. Plants.

[74] Kikiowo B., Ahmad I., Alade A.A., Ijatuyi T.T., Iwaloye O., Patel H.M. (2022). Molecular dynamics simulation and pharmacokinetics studies of ombuin and quercetin against human pancreatic α-amylase. J. Biomol. Stru. Dyn..

[75] Haque M.A., Hossain M.S., Ahmad I., Akbor M.A., Rahman A., Manir M.S., Patel H.M., Cho K.M. (2022). Unveiling chlorpyrifos mineralizing and tomato plant-growth activities of *Enterobacter* sp. strain HSTU-ASh6 using biochemical tests, field experiments, genomics, and in silico analyses. Front. Microbiol..

[76] Patel K.B., Mukherjee S., Bhatt H., Rajani D., Ahmad I., Patel H., Kumari P. (2022). Synthesis, docking, and biological investigations of new coumarin-piperazine hybrids as potential antibacterial and anticancer agents. J. Mol. Struc..

[77] Mathew B., Ravichandran V., Raghuraman S., Rangarajan T.M., Abdelgawad M.A., Ahmad I., Patel H.M., Kim H. (2022). Two dimensional-QSAR and molecular dynamics studies of a selected class of aldoxime- and hydroxy-functionalized chalcones as monoamine oxidase-B inhibitors. J. Biomol. Struc. Dynamics..

[78] Halder S.K., Ahmad I., Shathi J.F., Mim M.M., Hassan M.R., Jewel M.J.I., Dey P., Islam M.S., Patel H., Morshed M.R. (2022). A Comprehensive Study to Unleash the Putative Inhibitors of Serotype2 of Dengue Virus: Insights from an In Silico Structure-Based Drug Discovery. Mol. Biotech..

[79] Tivari S.R., Kokate S.V., Gayke M.S., Ahmad I., Patel H., Kumar S.G., Jadeja Y.S. (2022). A Series of Dipeptide Derivatives Containing (S)-5-Oxo-pyrrolidine-2-carboxilic Acid Conjugates: Design, Solid-Phase Peptide Synthesis, in vitro Biological Evolution, and Molecular Docking Studies. Chem. Select..

[80] Akintunde J.K., Akomolafe V.O., Taiwo O.A., Ahmad I., Patel H., Osifeso A., Ojo O.A. (2022). Antihypertensive activity of Roasted cashew nut in mixed petroleum fractions-induced hypertension: An in vivo and in silico approaches. Heliyon.

